# Assessment of HACCP plans and Colombian regulations in municipal cattle slaughterhouses for the assurance of standardised food safety and quality management systems

**DOI:** 10.1016/j.heliyon.2024.e40944

**Published:** 2024-12-05

**Authors:** José Fernando Solanilla-Duque, Sandra Morales-Velasco, Margarita del Rosario Salazar-Sánchez

**Affiliations:** Departamento de Agroindustria, Facultad de Ciencias Agrarias, Universidad Del Cauca, Sede Las Guacas, A.A, 190002, Popayán, Colombia

**Keywords:** Public health, Safety and quality, HACCP, Microbiological control, Processing, Meat supply chain, Health risk

## Abstract

Colombia has strengthened regulations to ensure standardized food safety and quality in bovine slaughterhouses. To this end, the Ministry of Health and Social Protection designed to empower INVIMA, as the governing and coordinating authority for the operation of the Official Meat Inspection, Surveillance and Control System. This regulation aims to evaluate the technical conditions of infrastructures, slaughter processes and quality and safety assurance systems. in the rationalization of public animal slaughtering facilities (PASFs). This study aims to promote easy-to-implement methodological practices to improve health and safety compliance in 47 cattle slaughterhouses. Sanitary profiles were designed based on the HACCP system and in accordance with Colombian regulations. Scores were established for the infrastructure, sanitary and environmental characteristics of the PASFs through the application of the Leopold Matrix as a monitoring system. The results indicate 32 % of slaughterhouses comply with minimum requirements, with significant deficiencies in equipment and waste management systems. There was evidence of scarce equipment, in addition, they do not have an efficient quality and safety management system to ensure the slaughter processes. Multivariate analysis revealed that 70–90 % of slaughterhouses fail to meet minimum standards established by Decree 1500 of 2007 and its modifications. It was shown that this methodology allows the establishment of a model for monitoring, follow-up, and assessment of compliance with the minimum requirements of HACCP plans and local regulations.

## Highlights

• Colombian norms prioritize improving PBAP's structure, safety, and environment.
• Implementing norms improves safety and reduces environmental impact.
• Constant regulation updates aid slaughterhouses infrastructure improvement.
• Risk reduction elevates PBAP's status to national distribution.
• Norm implementation raises PBAP staff's educational level.

## 1 Introduction

Livestock production is an important sector of global agriculture, providing a source of food, income, and employment for millions of people [1]. According to the Food and Agriculture Organization of the United Nations (FAO), the projected global livestock production in 2020 was 7.9 billion head, with cattle, pigs, sheep, and goats being the most common types of livestock, according to the U.S. Department of Agriculture (USDA) [[2], [3], [4]]. According to Statista Consumer Insights, it has reported that global meat production has been on an increasing trend over the last 7 years, rising from 317 million t in 2016 to 345 million t in 2022 [5]. However, by 2022, the Food and Agriculture Organization of the United Nations (FAO) projected that world meat production would reach a total of 361 million t, with a growth of 1.4 %, slower than the growth recorded in 2021 (4.5 %) [6]. However, according to the Organisation for Economic Cooperation and Development (OECD) and the FAO, world meat production by 2023 will increase by 19 % (57.7 million t) [6,7]. Of this increase, 78 % (45.1 million t) is accounted for by developing countries [8]. Similarly, the USDA forecast that global beef production will decline marginally this year to 59.2 million t, although production increases are expected in China, Brazil and Australia, which will be offset by declines in the United States and the European Union [9,10]. Similarly, it has forecast that world exports will decline 1.2 % by 2023 to 12.1 million t, 1 % less than this year, this decline has been attributed to lower demand from China. Also, the USDA has reported that global meat imports will fall by 2 % [11].

Colombia has an annual beef consumption of 176,442 t (in 1000 t carcass weight equivalent (CWE)) [5], ranking among the top 13 cattle producers worldwide, accounting for 2 % of the total and fourth place in Latin America as a producer of beef, with a growth in production of more than 22 % [12]. By 2020, beef consumption increased by 2 % (17.1 kg per person) [13,14]. However, beef intake has varied significantly, in 2014 beef had a consumption of 19.3 %, and in 2022 17.2 %; It is important to mention that the consumption preference of the population is beef for its taste. However, at the time of purchasing this protein, it becomes a high-priced product, which limits its accessibility [[15], [16], [17]].

In 2022, Colombia exported $64.7M in fresh or refrigerated beef, making it the 31st largest exporter of fresh or refrigerated beef in the world. In the same year, Meat of bovine animals, fresh or refrigerated was the 66th most exported product in Colombia. The main destination of Colombia's fresh or refrigerated beef and veal exports are Chile ($30.6M), Lebanon ($23.4M), United Arab Emirates ($4.02M), Saudi Arabia ($2.94M), and Jordan ($2.63M). The fastest growing export markets for Colombia's fresh or chilled beef and veal between 2021 and 2022 were Curaçao ($952k), Lebanon ($929k), and Maldives ($564) [18].

However, by 2023, Colombia's meat exports decreased by 42.3 % compared to 2022, explained by insufficient skilled labor in the processing and lack of sanitary practices to ensure the safety of the product. Thus, the Colombian Ministry of Agriculture, together with the Chinese sanitary authorities, obtained approval to export meat from Colombia to the Asian country, through the implementation of Hazard Analysis and Critical Control Point (HACCP) programs, with exports of 30,000 tons of meat, which together with the demands of other countries, from January to March 2024, recorded revenues of USD$ 20,068,621 million, strengthening the agro-industrial sector of the meat chain in the country [19].

Colombia has an annual beef production capacity of over 700 thousand tons. With approximately 29 million cattle, it is the fourth largest cattle herd in Latin America, with breeds of excellence such as the Cebu and Brahman. Additionally, the country has the potential to increase its capacity thanks to more than 10 million hectares with high aptitude for the commercial development of the beef industry [20].

The country's privileged geographical location allows cattle to be fed on pasture all year round, without the use of hormones or growth promoters and respecting the animal's natural cycle of development and fattening. 20 % of the beef produced in Colombia comes from animals slaughtered before 36 months of age, one of the requirements that foreign markets demand when buying high quality cuts or Premium nominees. Likewise, the country has a Sustainable Livestock Policy with a vision for 2050, which will enable the development of a sustainable livestock industry, leveraged on sustainability, quality and animal welfare [21].

Livestock production is therefore a key sector in the global and regional food industry, but it is also associated with risks of contamination by micro-organisms that are polluting and harmful to human health. Therefore, the Hazard Analysis and Critical Control Point (HACCP) system is becoming increasingly important to ensure the safety of animal products [22,23].

HACCP is a preventive food safety management system that identifies potential hazards, assesses risks, and implements control measures to prevent or minimise its occurrence. The system is based on seven principles: hazard analysis, identification of critical control points (CCPs), establishment of critical limits, monitoring of CCPs, corrective actions, verification and record keeping. In livestock production, HACCP is applied at various stages of the production chain, such as animal husbandry, feeding, transport, slaughter, processing, and distribution. For example, in pig farming, HACCP can be applied in the selection of feed ingredients, control of animal health and monitoring of hygiene practices. In meat processing, HACCP can be used to prevent cross-contamination, ensure proper cooking temperatures and control packaging and storage conditions [24,25].

Several countries have implemented HACCP-based regulations for livestock production. For example, in the European Union, HACCP is mandatory for all food businesses, including livestock production, under Regulation (EC) No. 852/2004. United States signed into law in 2011 the Food Safety Modernization Act (FSMA) was signed and enables the Food and Drug Administration (FDA) to better protect public health by strengthening the food safety system. This disposition requires, all food facilities, including those in the livestock production sector, to implement a HACCP based preventive control plan. The mandatory adoption of HACCP programs in Colombian plants, similar to the implementation in the European Union, could reduce microbiological risks, improving safety and quality in the national meat chain [26,27].

The application of HACCP in livestock production has been shown to improve food safety and reduce the risk of foodborne illness. A study in Turkey found that the application of HACCP in meat processing plants reduced the incidence of Salmonella contamination by 80 % (Buncic et al., 2014). Similarly, a study in Brazil reported a 30 % reduction of bacterial contamination in chicken meat following the implementation of HACCP-based controls [[28], [29], [30]].

From this data, it has been reported that 182 PASFs operate with bovine species, doing either only rendering, only deboning or both activities. In Colombia, these PASFs have simultaneous slaughter operations for both pigs and cattle. Of this total, only 132 slaughter plants are dedicated exclusively to cattle. On the other hand, of the 82 conditioning establishments, 71 are dedicated to preserving bovine carcasses. Of these, 55 work with bovine and porcine products, 4 with bovine only, 2 with bovine and poultry, 9 work with all 3 species and one has bovine, porcine, ovine, and caprine products.

Moreover, the identification of the environmental impact defined as the change or alteration of the environment, being a cause or an effect the mismanagement of a specific activity or inadequate human intervention, in this specific case slaughter plants. This impact can be positive when the result of an adequate implementation of a beneficial rule or measure to minimise risks and impacts to the environment or negative when a disruption of the ecological balance causes serious damage and impact to the environment. Furthermore, the measurement of environmental impact cannot be done with precision because the environment is a complex system. However, it is possible to make some estimates through the EIA (Environmental Impact Assessment), which emerged in the United States in the 1960s, and the respective Environmental Sustainability Reports (ESR), to try to minimise the negative impact. This work assessed the risks caused by poor infrastructure, inadequate sanitary management, and environmental impacts in 47 public animal processing plants (PASFs) due to deficiencies and non-compliance in the implementation of current regulations.

## 2 Materials and methods

### 2.1 Diagnosis and health profile of PASFs

Using checklists, a diagnosis of the conditions of each of the PASFs was made according to the criteria contemplated in the Hazard Analysis and Critical Control Points (HACCP) plan established by the FAO and Decree 539 of 2014. To determine the sanitary profile, 18 criteria were established, corresponding total 43 requirements with a maximum score of 715 points, equivalent to 100 % compliance with the requirements to assess the quality and safety criteria according to current regulations (Appendix 1).

### 2.2 Environmental impact assessment

For the identification of the different environmental impacts by the PASFs, the following was performed by means of the Leopold Matrix [31,32]. The relationship between actions and environmental factors was determined in terms of magnitude and importance (scale of 1–10) where 10 represents an unfavourable magnitude or a minor interaction and 1 a favourable magnitude. This double-entry matrix has as rows the environmental factors that can be affected and as columns the actions that take place and that can cause impacts [[33], [34], [35]]. Through the application of this tool, environmental impacts can be evaluated qualitatively and quantitatively, and thus establish an environmental management plan that involves preventive, corrective and mitigation actions, etc., focused on minimizing the identified environmental impacts. For the identification of the environmental quality of the area of influence of the assessment, a strict description of these was made to assess in each of the PASFs, with the objective of identifying the natural resources present and determining which environmental components are being affected.

### 2.3 Parameters and rating scales for the implementation of the Leopold Matrix

Once the environmental aspects of the area and the process stages in the PASFs had been identified, we proceeded to establish which environmental factors may be affected to form the rows and the actions that will take place and that may cause impacts, which make up the matrix, to identify the interactions that we intend to evaluate (Appendix 2). The processes are described, and the critical points of environmental impact generation are identified, using the Leopold matrix [36], a tool that qualifies the impacts that interrelates the activities of the PASF process with the different environmental components involved, weighting the Magnitude (Ma) of each environmental impact (Table 1) based on the weighting of the criteria of Character (C), Intensity (I), Extent (E) and Duration (D); and the Importance (Im) based on the weighting of the criteria of Risk (Ri) and Reversibility (R) [[33], [34], [35]].

**Table 1:** Rating assigned for each Magnitude parameter.

PARAMETER	VALUE ASSIGNMENT
WI (Intensity Criteria)	0,4
WE (Extend Criteria)	0,4
WD (Duration Criteria)	0,2

### 2.4 Magnitude (Ma)

The magnitude criterion relates the criteria of Character (C), Intensity (I), Extend (E) and Duration (D). This parameter is calculated through the following equation (see Eq. 1.):

1 *Ma* = *C*[(*IWI*) + (*EWE*) + (*DWD*)]

Where, C is the character of the impact, WI (Intensity Criteria), WE (Extend Criteria), WD (Duration Criteria) which determines whether the actions performed during the PASFs process positively (+) or negatively (−) affect the characteristics of the safety, infrastructure, and environmental components. I is the intensity, it assesses the strength of the impact caused by the project activities on the affected environmental component. The quantitative assessment of this parameter is 10.0 for high intensity, 5.0 for medium intensity and 2.5 for low intensity. E is the extend, which assesses the spatial influence of the expected impacts on the environment. The quantitative assessment of this parameter is 10.0 for a regional extent, i.e., when large areas are disturbed; 5.0 for a local extent, i.e., when areas in the immediate surroundings are disturbed; and 2.5 for a point extent, i.e., when a localised impact is involved. D is the duration of the action, which determines the permanence of the action on the environmental components analysed, i.e., it refers to the extent of time that the effect lasts and can be temporary, periodic or permanent, also considering the future or indirect implications (Appendix 3).

Table 2 shows the qualitative and quantitative rating scale for the parameters of character, intensity, extent, and duration. The sum of the parameters of intensity, extent and duration corresponds to 100 % of the assessment of the magnitude, weighting each parameter, assigning the weights found in Table 1. Once the magnitude of the impacts has been calculated, its qualitative assessment can be determined according to the following scale: from 7.6 to 10.0 very high magnitude, from 5.0 to 7.5 high magnitude, from 2.6 to 5.0 medium magnitude and from 1.0 to 2.5 low magnitude (Table 2).

**Table 2:** Qualitative and quantitative rating scales for character, intensity, extent, and duration parameters.

PARAMETER	RATING SCALE	
	Qualitative	Quantitative
**Character (C)**	Positive	1
	Negative	−1
**Intensity (I)**	Very High	10
	High	7,5
	Medium	5
	Low	2,5
**Extent (E)**	Regional	10
	Local	5
	Punctual	2,5
**Duration (D)**	Permanent	10
	Periodic	5
	Temporary	2,5
**Magnitude (Ma)**	Negative Very High	−10 to −7,6
	Negative High	−7,5 to −5,1
	Negative Medium	−5 to −2,6
	Negative Low	−2,5 to −1
	Positive Very High	+10 to +7,6
	Positive High	+7,5 to +5,1
	Positive Medium	+5 to +2,6
	Positive Low	+2,5 to +1
**Risk (Ri)**	Very High	10
	High	7,5
	Medium	5
	Low	2,5
**Reversibility (R)**	Irreversible	10
	Low Reversible	5
	Reversible	2,5
**Importance (Im)**	Very High	10 - 7,6
	High	7,5 - 5,1
	Medium	5 - 2,6
	Low	2,5 - 1

### 2.5 Importance (Im)

The significance criterion refers to the severity, transcendence, or degree of influence that the effect or impact of an action has on an environmental factor and relates the criteria of Risk (Ri) and Reversibility (R). This parameter is calculated through equation (2).

2 *Im* = (*RiWRi*)(*RWR*)

Where, W is criteria, Ri: risk, refers to the probability of occurrence of an effect that an action causes or will cause on the factor with which it interacts. The quantitative assessment of this parameter is 10.0 for high risk, 5.0 for medium risk and 2.5 for low risk. R: reversibility, which refers to the possibility of the environment to return to the original situation, i.e., it measures the capacity of the system to return to a situation of equilibrium similar or equivalent to the initial one.

The environmental impact caused is reversible if the original conditions reappear naturally or induced over time; and it is irreversible if the action of natural processes alone is not sufficient to restore the original conditions. Table 1 shows that the quantitative assessment of this parameter is 10.0 for an irreversible impact, 5.0 for a slightly reversible impact and 2.5 for a reversible impact. (Daza Serna et al., 2016). In equation (2), the sum of the risk and reversibility parameters corresponds to 100 % of the significance rating, weighting each parameter with the weights described in Table 1.

Once the identified impacts have been qualified and to have a general idea of its valuation, the result is calculated in such a way as to obtain the quantitative qualification of each effect on the criteria of magnitude and intensity, which will be measured by maximum positive values of +100 or negative values of −100, classified on the scale presented in Table 3. This table contains the impact degree codes by colour.

**Table 3:**
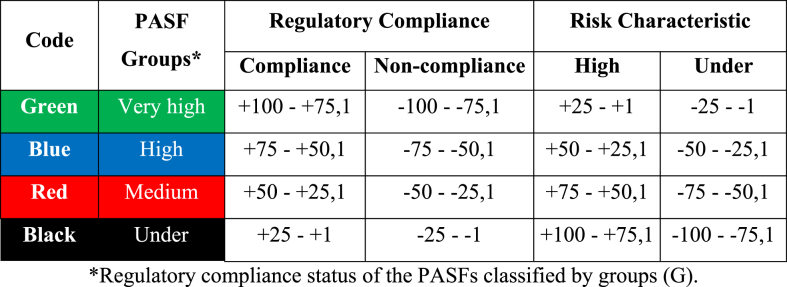
Scales and colour coding of impact classification of safety, infrastructure, and environmental characteristics, according to groups with low, medium, high and very high compliance according to regulatory compliance and the risk caused in the different characteristics assessed.

### 2.6 Implementation of the leopold matrix

The values were recorded by filling in the Leopold Matrix format, considering the following steps (Appendix 4).

a. In the format containing the Leopold matrix, the tabs corresponding to the Characterisation (C), Intensity (I), Extend (E), Duration (D), Risk (Ri) and Reversibility (R) of the impacts of the process must be filled in, intercepting those columns (actions or activities of the process) with the rows (environmental components affected), identifying the boxes where impacts occur.
b. Within the boxes with the identified impacts, the following parameters are evaluated: characterisation of the impacts, we establish a value of −1 if the environmental impact is negative or +1 if it is positive; to evaluate the intensity, a qualitative and quantitative rating scale is used according to the evaluator's criteria (high criteria = 10, medium = 5 and low = 2.5). The extent is rated depending on the scope of the impact, regional = 10, local = 5 and punctual = 2.5; to determine the duration of the impact, if it is temporary, it is assigned a rating of 2.5, if it is periodic it is rated at 5 and if it is permanent, it is assigned a value of 10. The probability of an impact occurring is assessed (high = 10, medium = 5 and low = 2.5). Finally, the reversibility of impacts is analysed as follows: Irreversible = 10, low reversible = 5 and reversible = 2.5.
c. Once the parameters C, I, E and D have been evaluated, the Magnitude (Ma) was calculated and with the values of Ri and R the Importance (Im) of the impact was calculated using equations 1, 2).
d. According to the Magnitude (Ma) and Importance (Im) obtained, the Magnitude or Importance matrix is filled in to facilitate the comparison of values and to highlight the significance of the environmental impacts according to the coding described in Table 3 (Appendix 5).

### 2.7 Statistical analysis

All observations obtained were analysed by multivariate statistical analysis to explain and predict the degree of relationship that exists between the levels of regulatory compliance of the different PASFs (weighted linear combination of the variables). In this way, an attempt was made to analyze the number of variables and the multiple combinations existing between the levels of compliance with infrastructure, safety and environmental impact (called categories) and the degree of regulatory compliance, i.e., the scores achieved (called groups). In this order of ideas, the scores in the same category were added together and normalized with respect to the maximum and minimum scores, thus obtaining the percentage of compliance per category in each municipality evaluated in terms of the degree of regulatory compliance.

The K-Means Cluster Analysis algorithm is applied to classify the requirements of the standardised categories [37]. K-means requires an input value for K, the number of clusters. The number of clusters can be determined empirically from organized the data. This algorithm uses minimum sum of squares to assign observations to groups (G). The clustering method allows the partitioning of a set of n elements into k groups (G) in which each element belongs to the group closest to the group mean (centroid). The elements are sorted in such a way that the value of the mean converges to the centroid. The algorithm reassigns the elements and recalculates the centroids in search of an optimal clustering. Each observation is allocated to the closest cluster, and the distance between an observation and a cluster is calculated from the Euclidean distance between the observation and the cluster centroid (OriginPro versión 2018).

The discriminant analysis (DA) model was used to determine a criterion that would allow us to decide to which group a certain individual belongs based on the region to which the PASFs belonged, from the information in the organized data, as described above [38]. Principal component analysis (PCA) was performed. This analysis is useful for reducing and interpreting large multivariate data sets with underlying linear structures. This multivariate analysis was considered to establish the variables that are highly correlated between the levels of compliance among the Categories and Groups established in this study [39]. The data were also analysed using partial squares (PLS). This multivariate analysis combines the features of PCA and multiple regression. First, it extracts a set of latent factors that explain as much of the covariance between the independent and dependent variables as possible. Next, a regression step predicts the values of the dependent variables using the decomposition of the independent variables. Useful to establish which of the highly correlated variables become independent variables [40]. The objective was to establish the correlation between the levels of compliance between the Categories and Groups established in this study according to the status.

This analysis was used to discriminate between three (3) groups of PASFs in terms of the levels of compliance with the categories (C) of infrastructure (I), safety (S) and environmental (E). Very high (G1), high (G2), medium (G3), and low (G4) compliance groups were established and the PASFs belonging to each group were classified.

## 3 Results and discussion

### 3.1 Governmental structure and regulations in Colombia

The distribution of livestock production is uneven across the world, with developing countries accounting for a larger share of the world's livestock population and production. For example, Asia has the largest number of livestock, with 44 % of global livestock production, followed by Africa (25 %) and the Americas (19 %) [7,41].

However, the environmental effects of livestock production are gaining increasing attention in recent years [42]. The world population is expected to reach 9.6 billion by 2050, and consequently, food production will have to double and agricultural infrastructure will have to increase, in all countries of the world, to meet the demands focused on food production. agricultural infrastructure, in all countries of the world, to meet the demands focused on meat consumption [[43], [44], [45]].

Therefore, ensuring hygiene in slaughter plants and meat processing is a governmental priority, and therefore, legislation must permit, prescribe, prohibit, and set targets in this economic activity [46]. Laws and administrative procedures are international, regional, or national jurisdictions. These legislations must guide and support the different principles, codes, guidelines, and agreements of the implemented regulations, which are supported by international provisions established by institutions such as the FAO, World Health Organisation (WHO), World Organisation for Animal Health (OIE), World Trade Organisation (WTO) and Codex Alimentarius [[47], [48], [49], [50], [51]].

In the inspection of food safety and security, specifically in meat, official control is important, both in slaughter plants as well as in retail outlets, this must be of high quality and its control must be conducted efficiently to guarantee the health of consumers and confidence in the animal slaughter plants. This inspection consists of monitoring and inspection of the food chain, ante-mortem, and post-mortem inspection of carcasses, which is intended to guarantee the safety of the meat, health, and welfare of the animals, as well as the prevention of diseases transmissible from animals to humans. In this context, Colombia had Decree 2278 of 1982 [52] (repealed by Article 98 of Decree 1500 of 2007 [53]), which partially regulated Title V of Law 09 of 1979 regarding the slaughter of animals for public slaughter for human consumption, processing, transport, and marketing of its meat [[52], [53], [54], [55], [56], [57], [58], [59], [60], [61], [62], [63], [64]].

In accordance with Decree 1500 of 2007 (as amended by Decree 2965 of 2008 [60]; Decrees 2380 [65], 4131 [66], 4974 [67] of 2009; Decree 3961 of 2011 [61]; Decrees 917 [68], 2270 [63] of 2012), the surveillance and control of the meat sector in Colombia is conducted by: (i) the Colombian Agricultural Institute (ICA), in charge of the prevention, surveillance and control of sanitary risks of food production from the agricultural, fisheries and aquaculture sectors to guarantee agri-food health and safety (Decree 4765 of 2008 [69]), is responsible for supervising animal processing plants, with the purpose of evaluating the quality of livestock products marketed in the different regions of Colombia and issuing the Certificate of Sanitary Inspection (CIS); (ii) the National Institute for the Surveillance of Medicines and Food (INVIMA), health authority in charge of the inspection, surveillance and control of the transformation in the meat production chain, in rendering plants, deboning, depressing and conditioning of meat and edible meat products (R2021043230 of 2021 [70], which establishes the procedure for obtaining the sanitary authorisation and registration, before the INVIMA) and (iii) the Departmental Health Secretariats, is the territorial entity responsible for fully evaluating the sanitary conditions and good manufacturing practices (GMP) of establishments dedicated to the storage, transport, distribution and sale of edible meat products and meat by-products at regional level (Decree 1500 of 2007, Decree 539 of 2014). Despite this governmental structure, processing plants in Colombia are deficient in four fundamental technical conditions: infrastructure, operational processes, environmental impact management and quality assurance systems. quality assurance systems [71]. In response to this, there is a legal framework that provides competent strategies for the meat sector chain. Many of these efforts to improve the legal structure for the surveillance and control of the conditions for the processing of animals for public slaughter in Colombia have not been sufficient due to the low level of investment in both the PASFs and the meat outlets for human consumption.

For this reason, the National Government implemented Decree 1500 of 2007 (modified by Decree 2965 of 2008 [60]; Decrees 2380 [65], 4131 [66], 4974 [67] of 2009; Decree 3961 of 2011 [61]; Decree 917 [68], Decree 2270 [63] of 2012) which established the technical regulations through which the Official System of Inspection, Surveillance and Control of Meat, Edible Meat Products and Meat Derivatives intended for human consumption was also created. This has been regulated by resolutions: R072 of 2007 [72] (adopts the Manual of Good Management Practices for the Production and obtaining of the Skin), R2905 of 2007 [73] (technical regulation on sanitary requirements and safety and the provisions for its processing, deboning, storage, marketing, sale, sale, transport, import or export), R18119 of 2007 [74] (regulating the requirements of the Gradual Compliance Plan and establishing the procedures for the processes of Registration, Sanitary Authorisation and Registration of these establishments), and its regulations R240 [75], R241 [76] and R242 [77] of 2013 that establish sanitary requirements for the operation of animal processing plants for public supply (PASF). From the above, the creation of Decree 2270 of 2012 [63] that modifies 13 articles of Decree 1500 of 2007 [53], which updates the Official System, mentioned above, throughout the national territory, stands out (*Decree 2270 of* 2012 [63]; *Resolution 3009 of 2010* [78]; *Resolution 2674 of 2013* [79]*; Resolution 3753 of 2013* [80]*; Resolution 2690 of 2015* [81]).

It is important to highlight that in the PASFs, the veterinary doctor supported by the sanitation technicians, both certified by INVIMA, are in charge of conducting food safety inspections and surveillance of the safety procedure, handling, conservation, transport of meat products and main zoonoses transmitted by animals, organs and waste in the slaughterhouses, as well as surveillance and control in transport vehicles and wholesale and retail meat outlets in Colombia. The purpose of these officials is to verify the control system in accordance with the current regulations. Therefore, the verification and implementation of the control system guarantees the hygiene of the process in slaughter plants, which is essential to maintain the safety of meat distribution and consumption [25,82,83]. Consequently, INVIMA was entrusted with the process of inspection, control, surveillance, and verification of compliance with the safety assurance system stipulated in Article 26 of Decree 1500 of 2007 [53]. When comparing the percentage of non-compliance with safety standards in Colombia with studies carried out in slaughter plants in Europe, as stated by Alvseike et al. [82], it is evident that they improve their hygiene conditions after implementing stricter controls. The findings on 66 % non-compliance in hygiene and sanitation practices are consistent with international studies, where similar conditions led to improvements in safety following the implementation of HACCP standards and staff training [84].

### 3.2 Policy implications

The lack of duly established Land Management Plans (LMP) has not allowed municipalities in Colombia, to implement an adequate sanitary and food safety culture. Which has not allowed the PASFs for cattle and pigs to be correctly located in accordance with the provisions of the regulations in force. Consequently, non-compliance with technical and environmental requirements and protocols established in the current regulations, and demanded by INVIMA, are evident in many of the country's PASF. In accordance with the above, the regulation established a gradual period for the PASFs to adapt its plants and comply with the standard, but the legislative provision was amended by Decree 2270 of 2012, giving a period of 3 and a half years for compliance with the current standard.

For this reason, the National Government implemented Decree 1282 of 2016 [64], which regulates the provisional sanitary authorisation issued by INVIMA. Consequently, PASFs that do not comply with the requirements of Decree 1500 of 2007 [53] (which entered into force in August 2016) and its amendments, depending on the regulations applicable to the corresponding species, will be subject to definitive closure by this entity and to the sanitary safety measures contemplated in article 576 of Law 9 of 1979 [54] and the respective sanctioning processes [64]. However, the requirement to present the Gradual Compliance Plan (GCP) or the presentation of Rationalization Plans for Public Animal Slaughtering Facilities (RPPASF), is a priority for the territorial entities, so that the PASFs comply and establish the appropriate quality assurance and safety systems, and also, implement the necessary requirements established in the current regulations to comply with HACCP prerequisites, implementation of sanitary standards, implementation of complementary verification programmes, product traceability, documentary records and sanitation standard operating procedures (SSOPs) (Appendix 6).

Currently, INVIMA, in the course of 2022, closed 40 establishments, and by January 2023, 5 more PASFs for a total of 409 closed establishments. Of these inspections and verification of requirements, 362 were PASFs and 47 conditioning establishments with cold rooms and operations related to cutting, washing, packing, and packaging of meat and meat products.

Consequently, following the efforts to apply the new regulations, the National Government has sought that the PASFs in Colombia certify compliance with 75 % of the requirements established in Decree 1500 and its amendments, and therefore, there are currently 443 authorised establishments, 172 establishments more than in June 2022. Of this total, 10 are closed, 171 correspond to processing only, 51 to processing and deboning, 47 to processing and deboning (of poultry), 65 to deboning only and 17 to deboning only. of bovine, porcine and poultry species under the guidelines of Decree 1500 of 2007 [53], Decree 2270 of 2012 [63] and the regulatory resolutions. The other 92 are approved conditioning establishments, of which 9 have voluntary closure and one (1) received an unfavourable concept for a total of 82 active establishments.

According to government reports, there are still 60 establishments that fall under Decree 1975 of 2019 [62]. However, as of September 2021, INVIMA issued Resolution 2021043230 [70] for those plants that comply with 75 % of the requirements of Decree 1500 to receive sanitary authorisation. Consequently, this action has contributed to reducing the number of slaughterhouses with provisional authorisation by 6 months.

### 3.3 Diagnosis and health profile of PASFs

**Fig. 1:**
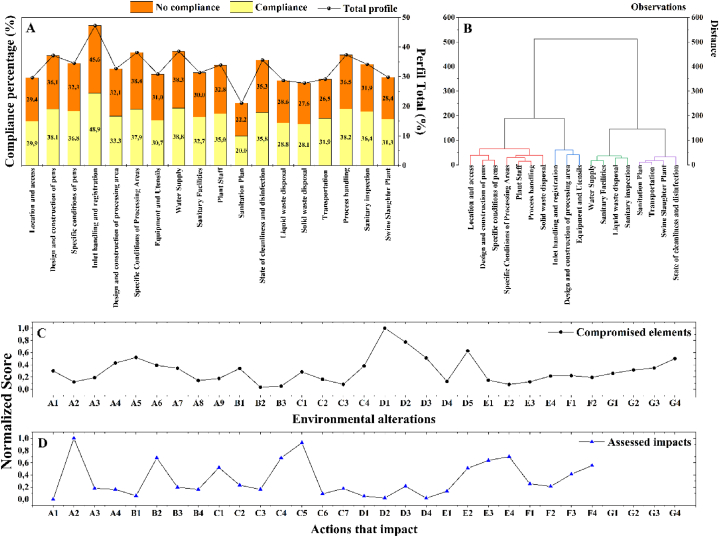
Sanitary profile of the PASFs, representing the percentage of compliance with the minimum requirements based on the current standard in force in Colombia (A and B). Environmental profiles based on the compromised elements and environmental alterations (C and D).

Fig. 1 show the sanitary profile (Fig. 1A y 1B) of the 47 PASFs, depicting the compliance level. Evaluation criteria were established in accordance with the prevailing standards in Colombia. Additionally, the environmental profile (Fig. 1C y 1D) was assessed by considering the compromised elements and environmental alterations of the 47 slaughter plants. In the evaluation conducted using the Leopold Matrix and Hazard Analysis and Critical Control Points (HACCP) and Hygiene and Quality Management (GHYCAL) checklists (Appendix 6), it was identified that 34 % comply with the minimum requirements demanded by the standard and the remaining 66 % do not comply with the management of GMP, infrastructure and adequate equipment for the processing process. It was established that 87 % are in urban areas and 52 % are located near water bodies, generating different environmental impacts. One of the particularities assessed was that 58 % are managed by public entities and 11 % are managed by private entities or mixed consortiums. However, 95 % of these PASF do not have an established and regulated maintenance programme with a slaughter frequency of 10 animals per week, which do not have wastewater treatment or solid waste management, being outside the provisions of Decree 1500 of 2007 (Fig. 1A).

According to the Land Management Plans (LMP) in force, more than 80 % of them must be relocated because they do not comply with the established regulations. In addition, 53 % operate at night, with the aggravating factor that 32 % cannot guarantee transport operations or supply chains for the distribution of the product (media and quarter channels), a situation that is largely due to the inefficient infrastructure investment in road networks by the municipalities. 80 % are supplied by the municipal aqueduct or non-potable water cisterns, more than 90 % do not have meters and only 64 % have a water storage system.

The dendrogram provides a hierarchical view of the similarity relationships between different aspects evaluated in the health profile of 47 slaughter plants. This analysis includes critical dimensions such as safety, quality, and operating conditions (Fig. 1B). In the structure of the Dendrogram, it is observed that each node or "junction point" in the dendrogram represents a specific component of the health profile. The height of the branches indicates the degree of similarity between plants based on these components; A shorter distance (height of the branches) implies a greater similarity in specific aspects of the health profiles. This allows us to identify plants that have common characteristics in terms of their management practices, which can indicate trends in compliance with regulations or shortcomings in some protocols.

In the identification of similarity patterns, it can be inferred that by examining the clusters in the dendrogram, patterns and correlations emerge that allow plants with similar health profiles to be grouped. The main clusters point to plant clusters that share strong characteristics in critical areas of safety and safety, while the subclusters identify more specific similarities, reflecting details in the implementation of protocols.

An example of Key Clusters can be seen on Floors 15 and 16, which show a strong correlation between these two plants in terms of their Transport and Sanitation Plan is highlighted by a low similarity distance (10.86953). This suggests that both plants manage these aspects in a comparable way, which may indicate a deficiency in the effective integration of these plans. This pattern of similarity could reflect structural issues in compliance with hygiene and transport standards, which are essential areas in the supply chain and safe food handling and could require improvements in planning and execution. However, on Floors 5 and 21, a low distance of similarity is observed in elements related to the Pig Slaughter Plant. This proximity may indicate a significant correlation in sanitation practices, suggesting a uniformity in certain sanitation practices between the two plants. This finding underscores the need for additional inspections to identify and address any gaps in specific hygiene procedures that could compromise safety.

On the other hand, in the design and construction of pens vs. specific conditions of the pens in Plants 2 and 3, a low distance of similarity (19.47407) was observed between these elements, highlighting a close relationship in the planning and the specific conditions of the pens in the evaluated plants. This suggests that the infrastructure and particular conditions of the pens in these facilities are strongly connected, likely reflecting a design that directly influences the animal handling environment, which is critical for stress reduction and improved sanitary conditions.

Regarding personnel and process management, elements 5 (Plant Staff) and 6 (Process handling) also have a low similarity distance, indicating a close connection in terms of personnel management and processing procedures in these plants. Additionally, plants 11 (Water Supply) and 12 (Sanitary Facilities) are grouped, showing a strong correlation between Water Supply and Sanitary Facilities. This finding underscores the importance of coordinating these two crucial aspects to maintain high sanitary standards. Current literature and regulations in Colombia on livestock facilities support the importance of proper design and specific conditions to ensure animal welfare and sanitary safety.

In this analysis, it is observed that "Inlet handling and registration," "Design and construction of processing area," and "Equipment and Utensils" form a group with distances of 60.88867 and 41.31798. These aspects are related, highlighting the importance of infrastructure and equipment in processing areas. These quality standards have been reported in various national and international regulations for the meat industry, supporting the relevance of these correlations for ensuring food safety.

On the other hand, "Liquid waste disposal" and "Sanitary inspection" are also grouped with a distance of 28.0116, suggesting that "Liquid waste disposal" and "Sanitary inspection" are interrelated. Literature on waste management in slaughterhouse plants supports the need for proper management to avoid sanitary risks. Upon deeper analysis, groupings and correlations were observed among plants that share similarities in these components. However, it is crucial to highlight that the dendrogram reveals not only positive correlations but also substantial differences between some slaughterhouse plants. For example, plants 29 and 30, grouped in the component related to the State of cleanliness and disinfection, show notable differences, suggesting significant variability in these specific areas between these plants.

Regarding the behaviour of the Clusters, it can be said that they allow prioritizing areas of intervention based on the similarity observed, that is, their application can be determined. An example of this can be explained in the sense that plants that share clusters in the dimension of "Design and Construction of Corrals" could benefit from maintenance programs and technical audits that guarantee an adequate environment. Otherwise, the clusters in the "Transport and Sanitation Plan" could be evaluated to establish specific guidelines that ensure safe and contaminant-free transport, minimizing the risk of cross-contamination and ensuring the quality of the final product. Therefore, the results of the dendrogram analysis not only help to identify common areas between plants, but also highlight management and operating patterns that may require specific attention, helping to optimize resources and reinforce critical protocols in slaughter plants to guarantee safety, quality of final products and mitigate environmental impacts.

In terms of sanitation and waste disposal, a strong connection is highlighted between location and access (1) and solid waste disposal (7) and liquid waste disposal (13). This suggests that proper location and access are correlated with efficient solid waste management, which can be crucial for maintaining high sanitary standards. Additionally, two significant groups with notably low similarity distances are identified: the group composed of elements 29, 30, 31, 32, 33, 34, and element 35, which is alone on a separate branch. These elements represent areas with marked differences in terms of sanitary compliance compared to the rest, with element 35 presenting the highest similarity distance compared to the others.

In terms of the most representative observations, those with significantly smaller distances between them are more similar. Plants grouped in the same cluster share closer characteristics in terms of their health profile. On the other hand, the unlabelled observation ("35″) is alone and at a considerable distance from the others, indicating a significant difference in its health profile.

In summary, this analysis highlights correlations between different aspects of the health profile of slaughterhouse plants. The most representative observations are those closely grouped, while the unlabelled observation stands out as unique in its profile. This correlation study provides valuable information for improving sanitary practices in these facilities, which is relevant in the field of food safety and management in different slaughterhouse plants based on their health, environmental, and infrastructure characteristics.

## 4 Infrastructural and environmental conditions as a function of the safety characteristic of PASF

It was found that 33 % do not comply with the minimum requirements for corrals because they have dirt floors, and only 2 % manage or implement a drainage system. 72 % of the plants do not quarantine the animals after its registration in the plant and only 14 % carry out external bathing, with manual washing predominating. On the other hand, it was established that only 5 % of the PASFs have a pig slaughter room, which is in poor condition and does not meet the minimum requirements of the standard. Consequently, it was found that 74 % do not have a programme for the management of biological vectors due to non-compliance with these minimum conditions. It should be noted that 63 % of the PASFs have animal management systems in minimum compliance conditions and that 92 % of these PASFs have cement floors in the corrals and management areas.

**Fig. 2:**
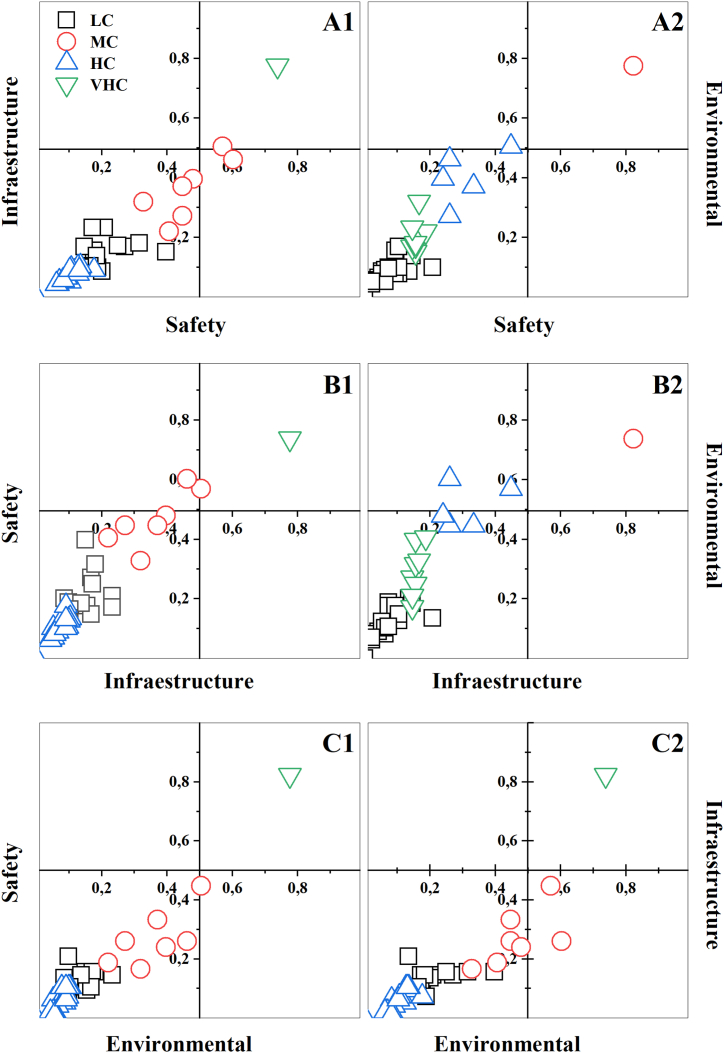
Correlation of the degree of impact between the implemented and installed conditions of the PASFs and the risks in terms of safety, infrastructure, and environmental impact characteristics at the time of the assessment using the "K-MEANS" algorithm to classify the requirements of the standardised categories.

Fig. 2 shows the correlation of the degree of impact between the implemented and installed conditions of the PASF and its risks in terms of safety, infrastructure, and environmental impact. The "k-means" classification method allowed the identification of CEBAPs in four groups corresponding to the very high (G1), high (G2), medium (G3) and low (G4) levels of compliance. It is evident that in the correlation of the degree of impact between the implemented and installed conditions of the FSAP and the risks based on the safety characteristics (CS), infrastructure (IC) and environmental impact (AC) at the time of the assessment (Fig. 2), in G1, there is only one CBAP that complies, corresponding to 2.1 %, degrees of compliance that match their centroid (CI = 73.8 %, CS = 77.6 %, and CA = 82.3 %). In the G2 group, 8 slaughter plants were identified (17 % of the CBAPs evaluated) with average compliance levels, represented by the centroid coordinates of 46 %, 35.7 % and 28.7 %, in the characteristics CI, CS and CA, respectively. Most of the plants are in the G3 and G4 groups, 38 in total, which correspond to 80.9 % of those evaluated, with coordinates of 15.4 %, 7.8 % and 5.2 %, in the characteristics CI, CS and CA, respectively.

It is evident that the CBAP belonging to G1 is located in the upper right quadrant, which corresponds to a good implementation of the regulations. However, the range of compliance with the CI category ranges from 32.8 % to 60.2 % for G2, and between 0 % and 31.6 % for G3 and G4, which shows the low compliance with this criterion in the other slaughter plants. Hence, there is a predominance of CBAP class III, IV and minimum, in accordance with Decree 1036 of 1991 [85]. Therefore, meat from these plants may only be marketed and consumed within the jurisdiction of the locality where it is located. Under these considerations, it was observed that most of the plants do not have a perimeter physical delimitation, which favors the appearance of pests and the entry of both personnel and vectors outside the beneficiation process. In addition, this study highlights possible risks, due to the presence of pathogens present in each of the processing stages, due to fecal contamination due to animal handling, infrastructure conditions and inadequate processing operations.

In this order of ideas, it was observed that 66 % of the slaughter and dressing operations are performed on the floor and only 34 % use an overhead mechanism, and generally, to carry out throat cutting and bleeding. Skinning is conducted manually, with only 5 % using a mechanical skinning system (Fig. 2).

In this analysis (Fig. 2), it was observed that the safety risk is largely due to non-compliance with the standard, as was observed in the airing operation where 95 % of the PASFs do not perform [86]. However, it should be noted that 26 % have a clearly defined evisceration area and 48 % clearly delimit this area with respect to the carcass quartering area. Moreover, 65 % have sanitary facilities in poor condition located within the internal area of the plant.

On average, only 5 % have the necessary equipment for slaughtering, preservation, or cold chain of meat and some PASFs have a solid and liquid waste management programme, showing that 6 % only have a biodigester for the treatment of its discharges. On the other hand, 99 % sell the blood-to-blood sausage producers or it is discharged directly into the sewage collectors, 1 % process the blood to obtain flour and white viscera for human consumption. Consequently, we observed that PASFs affect 72 % of the natural factors assessed with a high impact on public health, the surrounding community, and its surroundings, as shown in Fig. 2C1 and Fig. 2C2. Therefore, when the sanitary profiles were performed (Fig. 1A y Fig. 1B), the sanitary conditions are largely affected by the low degree of compliance with the requirements of the current regulations.

**Fig. 3:**
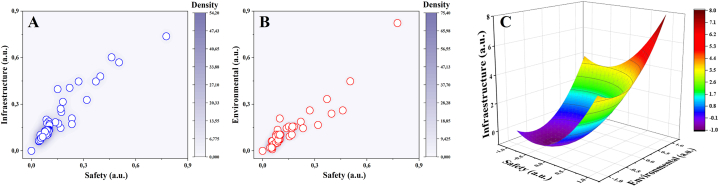
Density ratio showing the effect of non-compliance with safety conditions on the infrastructure characteristics (A) and environmental impact (B) of the surroundings of the PASFs using the Response Surface Analysis model (C).

This means that, in the PASFs, the risk of non-compliance is subject to the adequate implementation of the standard that guarantees the safety criteria in the processing of animals for public slaughter, as shown in Fig. 2A1 and Fig. 2A2, in which it is evident that it is deficient and that the density ratio of the degree of compliance (Fig. 3) is consistent with the behaviour of the data obtained analysed in the study. Consequently, it was observed that infrastructure and environmental impacts are strictly dependent on the lack of implementation of safety standards (Fig. 2B1 and Fig. 2B2).

The lack of adequate infrastructure in the PASFs, the lack of implementation of HACCP programs, the lack of implementation of standards that guarantee safety, and the lack of plans to mitigate the environmental impacts of the plants are the result of poor plant management, circumstances that are reflected in Fig. 2, with more than 50 % non-compliance in terms of the safety characteristic [26,27,87,88]. As an example of this situation, 67 % of the PASF do not carry out seizures of any kind and 33 % hand them over to the owners of the animals, deposit them in rivers or septic tanks, or, failing that, bury them. Only 4 % make seizures on the advice of the veterinary doctor [86,89].

This assessment made it possible to identify vulnerable elements of the environment (Fig. 3B), where the existence of aggressive actions was evidenced in 32 plants of the 47 municipal PASFs assessed (Fig. 3C). In this analysis of aggressive actions, the relationship of densities (Fig. 3) shows the effect of non-compliance with safety conditions in terms of infrastructure characteristics (Fig. 3A) and environmental impact (Fig. 3B) on the surroundings. It was observed in this correlation, that only 6 % of the PASFs have facilities and infrastructure in accordance with the standard and 4 % have facilities and infrastructure for the treatment of solid and liquid waste.

**Fig. 4:**
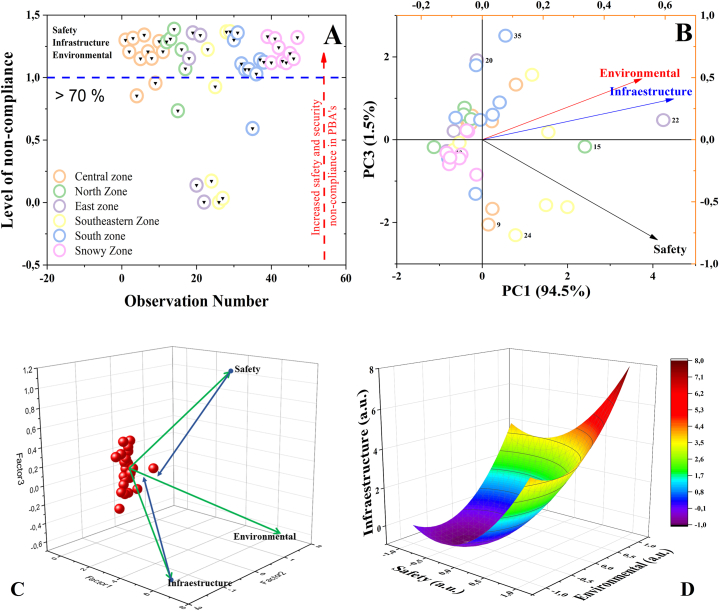
(A) Analysis of compliance level by regional stratification zones using Discriminant analysis model (DA). (B) Relationship of safety risk, infrastructure and environmental impact by regions using Principal Component Analysis (PCA) model. (C) Correlation of risk factors as a function of Safety, Environmental, Infrastructure characteristics using Partial Least Squares (PLS) model. (D) Effect on environmental conditions of PASFs using Response Surface Analysis model.

These observations are consistent with what was observed in the Discriminant Analysis (Fig. 4A), which according to what was recorded in the checklists individually and by region, it was recorded that 67 % do not have a wastewater treatment system and 29 % only have deficient measures to mitigate the environmental impact. Consequently, these waters are discharged directly into the sewage system and surrounding water bodies, behaviour that has been evidenced in the correlation of impacts shown in Fig. 2C1 and Fig. 2C2.

## 5 Environmental impact assessment

Continuing with the evaluation and focusing on the environmental impact in the 47 PASFs, it was recorded that only 8 % of these do not have a system for retaining heavy materials such as dung heaps and only a minimum percentage (6 %) provide satisfactory disposal of the solid waste generated. Regarding the monitoring of surfaces, utensils, equipment, and possible cross-contamination, it was recorded that cleaning and disinfection operations are performed with low concentrations of detergents and chlorinated products, which are disposed of as wastewater. Therefore, these results demonstrate that the level of compliance with regulatory requirements is low due to inadequate infrastructure and inadequate safety conditions, due to deficiency in the implemented cleaning and disinfection protocols established in the PASFs [90]. Hence, in previous decades, these conditions predominated in class IV, V and minimum requirements slaughterhouses, with no class I and II slaughterhouses in the department, according to the provisions of Decree 1036 of 1991 [85] which was repealed by Article 98 of Decree 1500 of 2007. In consideration of the regulations provided for in the latter Decree 1500 of 2007, these PASFs are now the ones that have the optimal conditions for operation, production, and that would possibly comply, in the last decade, with the requirements sought by the programmes undertaken by the departmental and national governments for the strengthening of the meat chain (Fig. 4B). In relation to this situation, it became evident that the analysis of the level of compliance by regional stratification zones conducted through Principal Component Analysis, the risk of safety, infrastructure and environmental impact are closely correlated with the risk factors according to the characteristics of Safety, Environment, Infrastructure that directly affect the conditions of environmental impact of the PASFs. In other words, implementing the standard to improve the safety characteristics and conditions significantly improves the characteristics and conditions of infrastructure and environmental impact in the PASFs (Fig. 4B). Otherwise, high risk is evident in the three characteristics evaluated, as shown in the Partial Least Squares Analysis, which reaffirms the behaviour of the PASFs according to the degree of compliance with the standard, showing that more than 70 % of the PASFs have a tendency towards negative impact. This study also highlights the existence of risk due to the possible presence of pathogenic microorganisms (*Salmonella* spp., *Escherichia coli* O157:H7*, Staphylococcus aureus, E. coli/Coliforms, Mesophilic aerobes* and *enterobacteria*), which are microbiological indices of safety and quality [[91], [92], [93], [94]].

This activity has generally been attributed to cross-contamination risk factors present in each and every stage of processing, due to the lack of delimitation between areas. Studies by other authors have reported that hygiene indicators, faecal contamination, microbiological safety, and shelf life are a function of deficiencies in cleaning and disinfection, process crossflow, operator crossflow, infrastructure and carcass handling [95]. Thus, the frequency of microbiological contamination transfer indicates that, on farm, vectors arise, especially attributed to faecal contamination, due to animal handling and infrastructure conditions of paddocks, stables, pens, and processing. The fact that most of the plants do not have a physical perimeter fence favours the appearance of pests and the entry of both personnel and vectors from outside production. On the other hand, cross-contamination can occur during the transport of the herd to the PASF as a consequence of the interaction between animals, the change of environmental conditions and the handling and hygiene in the vehicles. In addition, the infrastructure, handling, and process conditions allow considering the possible presence of pathogenic microorganisms linked to zoonotic vectors and food-borne diseases, both in live animals, environment, specific points of infrastructure in the plant and finally in the carcasses processed, which favours cross-contamination. A limited implementation and incorrect use of the aerial operation systems was observed as a critical point of contamination in the PASF process. Different authors refer that an adequate implementation of overhead rails in the process allows guaranteeing an adequate distribution between the carcasses and a safe distance between them and the floor, during its handling in the operation line, thus avoiding cross-contamination effects.

For the heat treatment of viscera such as stomachs and small and large intestines, the operations do not have adequate equipment or a plan for monitoring the temperatures applied in these processes, showing a deficient handling of the material and a possible high bacterial load. These observations agree with other studies which state that there is a high frequency of contamination by the transmission of pathogenic agents in the stages of elimination of legs, skin and viscera, in the scalding process and in the quartering operations, points in which there is a higher frequency of microbiological contamination than chemical contamination, due to its high microbial load and inadequate handling [96,97].

In addition, the environment in the operating areas can be a vehicle for contamination to other process steps such as in deboning, cutting, and deboning. Reported cases of Salmonella in slaughter plants in Switzerland and the United States, where non-compliance with regulations led to foodborne illness outbreaks, are strictly controlled and the responsibility of food regulators in each country. Therefore, non-compliance with sanitation and quality control plans increases these risks of microbiological contamination, as observed in Salmonella outbreaks associated with poor practices in slaughter plants in Switzerland and the USA [91,96,98]. Some authors have shown that lack of compliance affects the durability of meat products and increases the risk of antimicrobial resistance [86,89]. Consequently, the lack of effective control in the supply chain could increase this antimicrobial resistance in foodborne pathogens, a significant problem for public health regulatory entities, which studies such as that of Ferri et al. [86], have associated with poor sanitary conditions in the meat industry.

The inadequate wastewater management in the PASFs allows the identification of a potential *Salmonella* outbreak (data not shown), a risk that is imminent under the conditions evaluated in this study. Thus, cases of meningitis, related to *Streptococcus suis* in pork, outbreaks of salmonellosis and gastroenteritis, associated with *Salmonella* spp. in beef and chicken, can occur in PASFs, generating public health risks [24,99,100].

Although about 75 % of the plants are assisted by a veterinary doctor, he or she is not in charge of all the activities that fall under his or her responsibility, as external personnel or operators without proper training carry out processes such as heat treatment, preservation, and storage without carrying out any control or relevant sanitary registration. This role of veterinary staff is of great relevance as its functions contribute to creating a safe and secure system throughout the processing stages [89,101].

Residues such as blood and skins constitute a critical point of contamination in the process transferable to the carcass. Likewise, poor hygiene and cleanliness of facilities and poor cold chain management are points that favour the recontamination of carcasses, generally favoured by the bad behaviour of the operators, its low level of training and the lack of knowledge of the processes on its parts [25].

The alignment between HACCP plans and Colombian regulations in PASFs was evaluated, focusing on the standardization of quality management and food safety systems. Fig. 4C show a three-dimensional partial least squares (PLS) representation to analyze key factors such as food safety, environmental compliance, and infrastructure. The clustering of red points indicates consistent observations, reflecting homogeneity in regulatory compliance or non-compliance and minimal variability across the facilities assessed.

The direction and magnitude of the vectors reveal that food safety is the primary factor influencing operational practices, extending along Factor 3, while infrastructure and environmental aspects showed moderate contributions with low compliance levels. This suggests that HACCP plans are insufficient in most slaughterhouses, failing to adequately ensure food safety within these facilities. Additionally, infrastructure and environmental compliance were found to be substandard, requiring greater attention.

The convergence of data points towards the origin highlights the consistency of normative non-compliance across most plants, although outliers suggest accordance in systems with higher levels of compliance. These findings provide valuable insights for optimizing slaughterhouse practices, ensuring adherence to regulations, and strengthening food safety protocols in line with international standards.

## 6 Final considerations of the case study

In accordance with the above, in Colombia it has been necessary to implement the HACCP system on a mandatory basis (Decree 60 de 2002 [102]) as has been done in other countries, through the implementation of methodologies and technologies that guarantee optimal conditions in the processes to ensure public health in the food industries (Appendix 6). To this end, the National Government issued Decree 2965 of 2008, which regulates that all processing plants must ensure that its practices and processes guarantee the consumer a high-quality food free of contamination risks without underestimating the importance of the preservation of natural resources, with proper management of solid waste and the relevant control of emissions generated in its processes.

However, under the current conditions of the municipal PASFs evaluated, these regulations are limited due to the low budgets and resources allocated for this purpose. It is hoped that in Colombia, under the expectation of high competitiveness and the signing of free trade agreements, modernization policies can be applied to the slaughter of animals for public slaughter [103].

**Fig. 5:**
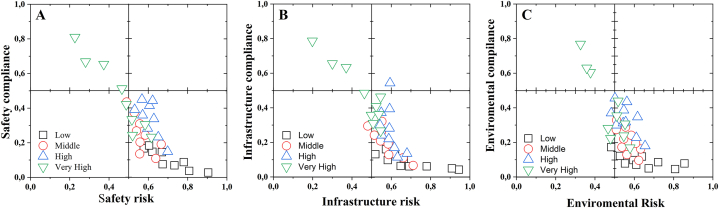
Correlation between compliance criteria and risk factors for safety, infrastructure and environmental characteristics assessment using the "K-MEANS" algorithm to classify the requirements of the standardised categories.

Fig. 5 shows the correlation between compliance criteria and risk factors in the assessed characteristics of safety, infrastructure, and environmental impact. It was found that most of the PASFs have high risk in the characteristics of safety, infrastructure, and environmental impact. It was also observed that around 6 % of the PASFs decreased its risk by at least 40 % in these three evaluated characteristics (Fig. 5A, B, 5C). This behaviour was due to an increase in the implementation of the current compliance standard by more than 60 %. This affirms that individual efforts in resource investment and implementation of the standard have been efficient and effective in some of these plants. These observations are in agreement with what is reported in Fig. 3, where the density ratios show the effect of non-compliance with safety and infrastructure conditions on the environmental impact of the surroundings of the PASFs. This shows that by implementing the standard in terms of improving the safety conditions in the plant, an improvement in the other two characteristics was evidenced, therefore, it is evident that the obligation to improve the infrastructure of the PASF is subject to the implementation of HACCP prerequisites and other regulations related to quality assurance and safety in plants, as shown in Fig. 4C. Where it was observed that having a deficiency in the infrastructure increases the environmental impact (Fig. 4A) and that by implementing the current standards the safety conditions and infrastructure decreases the risk for the PASF implementing the standards (Fig. 4D) [89,103].

In the analysis of the environmental impact in slaughter plants (PASFs), several types of specific pollutants and poor waste management practices were identified that generate significant effects on the environment. the use of the Leopold Matrix made it possible to evaluate the impacts caused by the processes of slaughter and handling of products of animal origin on the key environmental components (Appendix 1).

The specific pollutants identified are three important criteria, including contaminated wastewater, solid and organic waste, and air emissions. Consequently, the first criterion focuses on the discharge of wastewater without adequate treatment, rich in organic matter and pathogens (possibly Salmonella spp., *Escherichia coli*), has a negative impact on nearby water bodies. These waters often contain high levels of suspended solids, nutrients and blood residues that contribute to eutrophication and the proliferation of pathogenic bacteria, putting both human health and aquatic biodiversity at risk.

The second criterion, solid and organic waste, focuses on those generated, such as viscera, blood and skins. These are often stored without appropriate management measures, increasing the risk of contamination by leachate in the soil, and possibly the spread of zoonotic diseases. Some waste was found to be dumped directly into the sewer system or used for other by-products without proper sanitary controls, intensifying environmental and public health risks.

The third criterion refers to waste incineration operations, including the burning of animal remains, which emit polluting gases and bad odours, which affect air quality in adjacent areas. Odours represent a major problem in terms of the well-being of local communities due to the lack of updated Land Use Plans (LUP).

On the other hand, the poor management of waste identified are three criteria that are explained below:

Lack of wastewater treatment. Most PASFs do not have adequate wastewater treatment systems. Direct discharges into the environment and untreated bodies of water contribute to bacterial contamination and nutrient accumulation, altering local ecosystems and affecting the water supply of nearby communities.

Inadequate disposal of solid waste. The disposal of solid waste (such as organic remains and unused by-products) lacks adequate containment systems. Only a minority of PASFs have biodigesters or infrastructure for the treatment of these wastes, resulting in inappropriate disposal in rivers, septic tanks or burial without the necessary control measures.

Deficient separation and management of biological and non-biological waste. The lack of clear protocols for the segregation and handling of hazardous waste, such as contaminated materials and non-edible by-products, has facilitated cross-contamination and increased pathogen risks in processing and storage areas. This analysis highlights the need to adopt stricter waste management practices and to implement water and waste treatment technologies in PASFs, with the aim of reducing negative effects on the environment and improving the sustainability of the sector.

The general panorama of the PASFs of municipalities with populations of less than 20,000 inhabitants showed low economic development and a low level of specialised personnel linked to food quality and safety. Despite the efforts of the National Government to improve the conditions of this sector, there is still a deficiency in the adequate implementation of the regulations in force in an effective way by the territorial entities and these municipal PASFs due to the lack of investment resources to guarantee efficiency in the processing of animals for public slaughter. The increase of slaughterhouses in the national territory stands out, and with respect to the PASFs evaluated, a significant effort (6 %) was evidenced in compliance with the standard to reach the highest category (national distribution and export of meat products). Consequently, the Colombian National Government has committed to a dynamic and continuous renewal of the legal and regulatory support to optimize resources and implement processes to rationalise the PASFs, both for animal processing plants in the self-consumption category (plant authorised by the INVIMA, responsible for supplying meat to the respective municipalities) and animal processing plants in the national category (plant authorised by the INVIMA, responsible for the export of meat products) (Decree 2270 of 2012), which require this type of study to maintain its current operating status, continuous improvement of its facilities and equipment, as well as its sanitary conditions to comply with the quality assurance and safety plans for its products.

Consequently, the study includes a limited number of slaughter plants (47 plants), which may not be representative of the general situation in Colombia or in other regions with similar sanitary structures and operations. However, evaluations are influenced by the availability of information in each plant and by the conditions of infrastructure and equipment. In addition, there may be variability in inspection and enforcement practices between plants due to economic and geographical factors, which could lead to biases in the results. The application of non-destructive technologies such as infrared spectroscopy, freshness index (K index) by HPLC, imaging techniques, among other techniques [104,105], can allow to monitor meat quality in real time, increasing regulatory implementation to improve quality assessment in slaughter plants. The use of sensors could also be implemented to detect pathogens at critical points in the process, improving precision in sanitary control.

Future studies could incorporate more detailed environmental impact assessments using spatial analysis tools and geographic data modelling to determine patterns of pollution and impact on the natural environment [31,33]. It is recommended to explore the effectiveness of advanced GMP and HACCP evaluation methods in reducing microbial contamination in real time, using continuous monitoring technologies such as IoT, which can reinforce safety programs and improve product traceability.

## 7 Conclusions

The study revealed that approximately 66 % of slaughter plants in Colombia do not meet the minimum quality and safety requirements according to current regulations. This suggests that there is a significant gap in compliance with Hazard Analysis and Critical Control Points (HACCP) plans and in the implementation of proper waste management and environmental management practices. The application of the Leopold matrix made it possible to identify the areas of greatest environmental impact and risk in the sanitary profile of the plants, highlighting the deficiencies in infrastructure and solid and liquid waste management. These findings are crucial as they highlight the public health risks associated with non-compliance with these standards, underscoring the need to strengthen inspection protocols and management capacities in these facilities. It is necessary to strengthen the training of personnel in hygiene, waste management and maintenance of facilities. This would help minimise the risks of cross-contamination and improve sanitation practices in slaughter plants. Reinforce infrastructure in critical areas such as corrals, drainage systems, and equipment for proper waste management. Invest in wastewater treatment technology and monitoring systems to meet the requirements of current regulations. It is recommended to carry out regular monitoring and compliance audits using analysis tools that allow monitoring the degree of compliance in slaughter plants to identify high-risk areas and prioritize interventions or mitigations as appropriate. Given that there are differences between plants in terms of their compliance profiles, future research could focus on identifying regional barriers to compliance and proposing specific interventions based on international regulations and standards. Consequently, a national policy focused on the modernization of slaughter plants in rural areas of Colombia could improve compliance conditions in these facilities. This could include subsidies or tax incentives for plants to invest in infrastructure and in the implementation of HACCP standards. On the other hand, it is proposed to strengthen inspection programs in low-compliance slaughter plants, prioritizing areas that represent the greatest risk to public and environmental health, such as wastewater management and solid waste disposal. Generate investment plans that tend to implement training and education programs for personnel in slaughter plants in small municipalities. This would strengthen regulatory compliance and increase the safety of animal products. In conclusion, this study highlights the urgency of optimizing quality control and safety systems in slaughter plants in Colombia, promoting changes in infrastructure, training, and regulation to reduce public health risks and improve environmental sustainability. The proposed recommendations have the potential to raise health standards, minimizing the risks of foodborne illness and ensuring a safe supply of meat products for the population.

## CRediT authorship contribution statement

**José Fernando Solanilla-Duque:** Writing – review & editing, Writing – original draft, Validation, Methodology, Investigation, Formal analysis, Data curation, Conceptualization. **Sandra Morales Velasco:** Writing – review & editing, Validation, Supervision, Data curation. **Margarita del Rosario Salazar-Sánchez:** Writing – review & editing, Validation, Supervision, Data curation.

## Ethics statement

This study did not require review or approval by an ethics committee, as it solely involved the inspection and evaluation of compliance with HACCP system requirements and Colombian regulations regarding slaughterhouse activities. The study did not perform any slaughtering operations; it was strictly limited to verifying regulatory compliance within the facilities without engaging in the animal slaughter process. The evaluations were conducted with the supervision of officials from regulatory institutions, including the Agricultural and Environmental Prosecutor's Office and INVIMA. All assessments adhered to current Colombian regulations, as detailed in the manuscript.

## Data availability statement

Data will be made available on request.

## Funding sources

This research did not receive any specific grant from funding agencies in the public, commercial, or not-for-profit sectors.

## Declaration of competing interest

The authors declare the following financial interests/personal relationships which may be considered as potential competing interests: Declarations of interest: none.

## Footnotes

Appendix A Supplementary data to this article can be found online at https://doi.org/10.1016/j.heliyon.2024.e40944.
