# Supplementary material for: Assessment of HACCP plans and Colombian regulations in municipal cattle slaughterhouses for the assurance of standardised food safety and quality management systems
Source: Heliyon. 2024 Dec 5;10(24):e40944. doi: 10.1016/j.heliyon.2024.e40944 (PMC11698929; doi:10.1016/j.heliyon.2024.e40944)
Supplement: Multimedia component 2 [file mmc2.docx]

| **Category** | **Criteria** | N.R. | P. M |
| --- | --- | --- | --- |
| Infrastructure | Location and access | 10 | 50 |
|  | Design and construction. of corrals | 8 | 40 |
|  | Spec Conditions of corrals | 9 | 45 |
|  | Entry and Registration Management | 11 | 55 |
|  | Design & Construction Benefit Area | 14 | 70 |
|  | Spec Conditions of Benefit Areas | 9 | 45 |
|  | **Total Score** | **61** | **305** |
| Sanitary | Equipment & Utensils | 17 | 85 |
|  | Sanitary facilities | 6 | 30 |
|  | Plant Personnel | 10 | 50 |
|  | Sanitation plan | 3 | 15 |
|  | Cleaning and disinfection status | 3 | 15 |
|  | Transport | 2 | 10 |
|  | Process Management | 9 | 45 |
|  | Sanitary Inspection | 7 | 35 |
|  | Pig Slaughter Plant | 1 | 5 |
|  | **Total Score** | **58** | **290** |
| Environment | Water Supply | 5 | 25 |
|  | Disposal of liquid waste | 6 | 30 |
|  | Solid Waste Disposal | 13 | 65 |
|  | **Total Score** | **24** | **120** |
| **TOTAL SCORE** | | 143 | 715 |

Appendix 1. Description of the scores of the requirements evaluated in the grouping of the criteria into the categories CI, CS and CA.
