# Supplementary material for: Assessment of HACCP plans and Colombian regulations in municipal cattle slaughterhouses for the assurance of standardised food safety and quality management systems
Source: Heliyon. 2024 Dec 5;10(24):e40944. doi: 10.1016/j.heliyon.2024.e40944 (PMC11698929; doi:10.1016/j.heliyon.2024.e40944)
Supplement: Multimedia component 4 [file mmc4.pptx]

## Slide 1
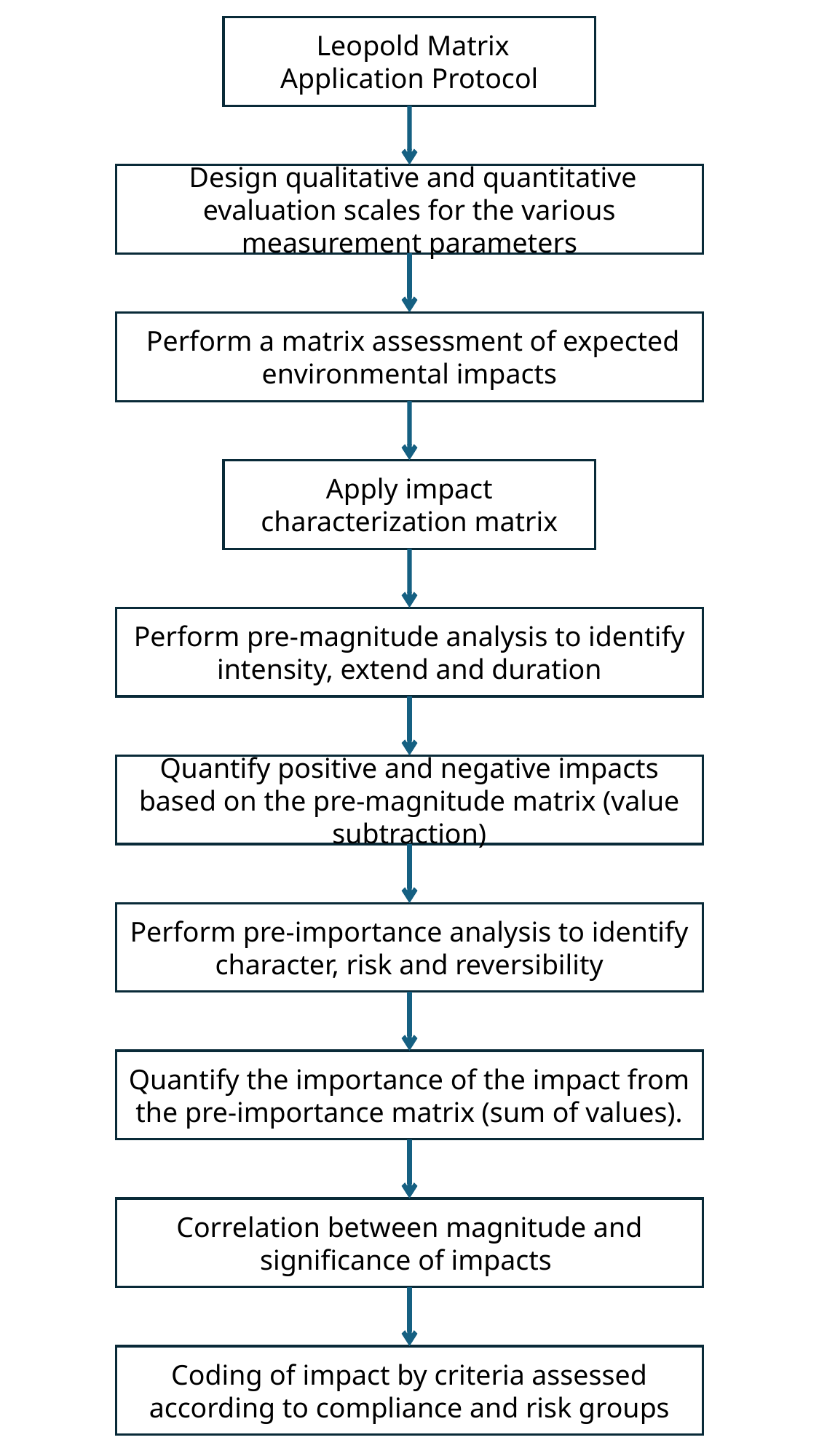

Leopold Matrix Application Protocol
 Design qualitative and quantitative evaluation scales for the various measurement parameters
 Perform a matrix assessment of expected environmental impacts
Apply impact characterization matrix
Perform pre-magnitude analysis to identify intensity, extend and duration
Quantify positive and negative impacts based on the pre-magnitude matrix (value subtraction)
Perform pre-importance analysis to identify character, risk and reversibility
Quantify the importance of the impact from the pre-importance matrix (sum of values).
Correlation between magnitude and significance of impacts
Coding of impact by criteria assessed according to compliance and risk groups
