# Supplementary material for: Assessment of HACCP plans and Colombian regulations in municipal cattle slaughterhouses for the assurance of standardised food safety and quality management systems
Source: Heliyon. 2024 Dec 5;10(24):e40944. doi: 10.1016/j.heliyon.2024.e40944 (PMC11698929; doi:10.1016/j.heliyon.2024.e40944)
Supplement: Multimedia component 5 [file mmc5.docx]

**Preliminary conditions**

The Leopold Matrix was used to evaluate the impact of three characteristics of the slaughter plants (technical conditions): infrastructure, slaughter processes, and quality assurance systems. This matrix made it possible to identify, qualify and weigh the impacts generated by the plants in the environment of influence. The structure of the matrix was organized in such a way that the elements involved, where environmental alterations are evident, were in the rows and the factors or actions that potentially impact the environment were in the columns, facilitating the identification of interactions based on the operability of the evaluated plant. The weighting and scoring system is key to quantifying the degree of impact, therefore, we proceed to give a brief explanation of how we develop it. However, it is important to consider the path schema for the application of the matrix.

**
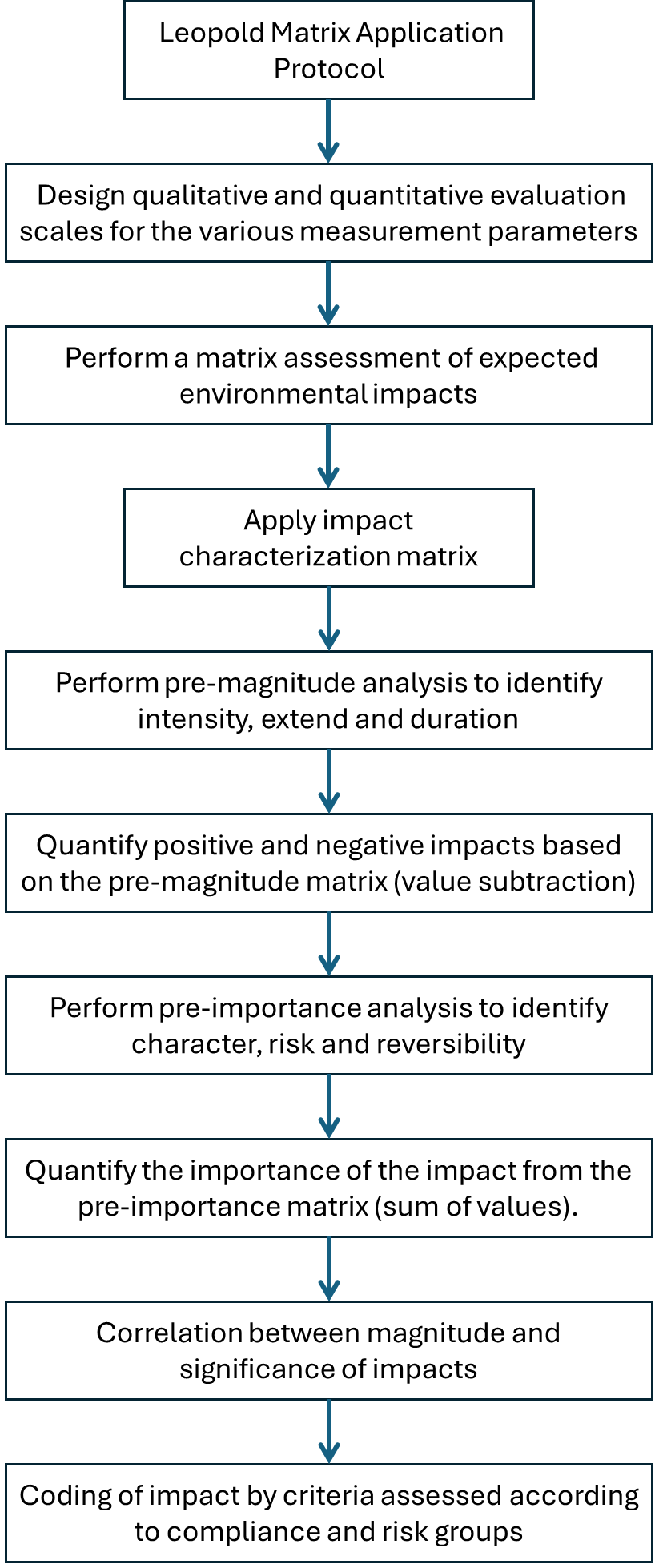
**

**Weighting and scoring system carried out with the data obtained from the application of the Leopold Matrix:**

1. **Assignment of Importance:**
   - Each environmental alteration and action was weighted based on its relevance to each plant based on the standard (according to current regulations in Colombia and international standards HACCP and GHYCAL). This weighting is usually represented on a scale of 1 to 10 (or sometimes in intervals of 1 to 5), where higher values indicate greater relevance or vulnerability of the affected or compromised element.
2. **Impact Score:**

- For each intersection (environmental alteration or impact action), scores were assigned in two dimensions:
  - **Magnitude**: it is the one that represents the intensity of the impact (for example, on a scale of -5 to 5), where negative values indicate adverse impacts and positive values indicate benefits. A value of 0 means no impact (see scoreboard).
  - **Significance**: This indicates the significance or degree of impact in the context of the process evaluated in the plant (e.g., on a scale of 1 to 10). The frequency and duration of the impact was also considered, in some cases, because some plants had little infrastructure or was in the process of improving infrastructure.

1. **Total impact calculation:**

- The final score in each cell was obtained by multiplying the magnitude of the impact by the importance. For example, if the magnitude of an impact is -3 and the importance is 8, the score would be −3×8=−24.

1. **Normalization of Scores**

However, since the Leopold matrix can generate a large number of scores due to multiple interactions, normalization of the data should make it easier to interpret the impacts. Therefore, normalization was performed to obtain scores on a uniform scale. This made it possible to compare impacts in different dimensions and improve the global understanding of environmental alterations (effects or compromised components). The data normalization process is described below:

- 1. **Identify the total range of impact:**
- Calculate the maximum and minimum value obtained in the complete matrix (considering all products of magnitude by importance).
  1. **Linear Scaling:**
- A standardized scale, e.g. from -100 to 100, was used to adjust the values. The normalized score (Pn) of each cell was calculated using the following equation:

$$P_{n}\left( \frac{\left( P-P_{min} \right)}{P_{max}-P_{min}} \right)X100-100$$

where:

- - P is the net score, i.e. score given by the evaluator (e.g. from 1 to 25)
  - Pmin is the minimum score, i.e. lowest possible score (e.g. 1)
  - Pmax is the maximum score, i.e. highest possible score (e.g. 25)
  - This method converts the value of each cell to a scale of -100 to 100, making it easier to compare between impacts.

We have simplified the normalization of these scores by following the following procedure for a better understanding of the normalization process:

1. **Calculate the Maximum Total Score:**The theoretical maximum score is determined, which would be the magnitude and maximum importance (10 and 10 respectively) multiplied by the number of possible interactions.
2. **Divide each Score by the Maximum Score**:
    Then divide the score of each cell by the theoretical maximum score and multiply it by 100 to obtain a percentage.

$$Impacto Normalizado=\frac{Puntaje de impacto}{Puntaje total máximo} x 100$$

This allows each impact to be expressed on a scale of 0 to 100, making it easier to prioritise environmental impacts and make decisions more clearly and concisely.

This system made it easier for evaluators and decision-makers to identify the critical impacts and generate respective mitigation actions on the consequences produced by the actions that impact the environmental components. The objective of these results was to generate individually, for each plant, the different strategies to minimize the negative effect on the environment. These are not included in the work for reasons of leave by companies.

1. **Final Consideration**

- The normalized values made it possible to identify which activities have the greatest impact (positive or negative), allowing decisions to be made for the mitigation or reinforcement of impacts according to the analysis of each plant and their respective environmental interactions.
- This approach helps to provide an objective and standardized view of environmental impact, facilitating decision-making based on clear and standardized data.
