# Supplementary material for: Assessment of HACCP plans and Colombian regulations in municipal cattle slaughterhouses for the assurance of standardised food safety and quality management systems
Source: Heliyon. 2024 Dec 5;10(24):e40944. doi: 10.1016/j.heliyon.2024.e40944 (PMC11698929; doi:10.1016/j.heliyon.2024.e40944)
Supplement: Multimedia component 6 [file mmc6.docx]

Appendix 5.1. Aspects and number of elements evaluated for national category animal processing plants based on current regulations and HACCP system

| Evaluated aspect | | Number of Items |
| --- | --- | --- |
| 1 | Location and access | 4 |
| 2 | Design and build | 17 |
| 3 | Drainage Systems | 7 |
| 4 | Ventilation | 3 |
| 5 | Lighting | 3 |
| 6 | Sanitary facilities | 20 |
| 7 | Integrated Pest Control | 1 |
| 8 | Liquid and solid waste management: | 4 |
| 9 | Water quality | 8 |
| 10 | Healthcare Operations | 1 |
| 11 | Manipulative personnel: | 29 |
| 12 | Facilities, equipment and utensils | 2 |
| 13 | Entrance area | 6 |
| 14 | Corral Area: | 23 |
| 15 | Slaughter and slaughter room | 1 |
| 16 | Desensitization, indentation, and intermediate or processing area. | 38 |
| 17 | Termination and Exit Area | 1 |
| 18 | Carcass conditioning area | 7 |
| 19 | Refrigeration, freezing and storage rooms. | 22 |
| 20 | Posting area. | 12 |
| 21 | Dispatch area | 8 |
| 22 | Other facilities. | 12 |
| 23 | Sanitation Standard Operating Procedures (SOPs) | 11 |
| 24 | Complementary programs | 8 |
| 25 | Inspección ante-mortem y post-mortem | 29 |
| 26 | Transport guide | 1 |
| 27 | Sampling Plan | 8 |
| 28 | Cold chain and storage of meat and meat products. | 3 |
| 29 | Shelf life of meat and edible meat products. | 1 |
| Total number of items evaluated | | 290 |

Appendix 5.2. Aspects and number of items evaluated for animal processing plants in the self-consumption category based on current regulations and HACCP system

| Evaluated aspect | | Number of Items |
| --- | --- | --- |
| 1 | Invima approval. | 1 |
| 2 | Location and access | 3 |
| 3 | Design and build | 17 |
| 4 | Drainage Systems | 4 |
| 5 | Ventilation | 3 |
| 6 | Lighting | 2 |
| 7 | Sanitary facilities | 5 |
| 8 | Sanitary filters: | 2 |
| 9 | Integrated Pest Control | 1 |
| 10 | Liquid and solid waste management | 2 |
| 11 | Water quality | 4 |
| 12 | Healthcare Operations | 1 |
| 13 | Handling staff | 3 |
| 14 | Hygienic practices and protective measures | 19 |
| 15 | Facilities, equipment and utensils | 2 |
| 16 | Entrance area | 2 |
| 17 | Corral area | 7 |
| 18 | Specific requirements | 4 |
| 19 | Requirements for Operations | 2 |
| 20 | Profit Room | 4 |
| 21 | Desensitization and indentation section | 3 |
| 22 | Requirements of operations | 9 |
| 23 | Intermediate or processing section | 5 |
| 24 | Cattle processing operations | 15 |
| 25 | Termination and Output Section | 11 |
| 26 | Cooling System | 16 |
| 27 | Posting area. | 4 |
| 28 | Other facilities. | 4 |
| 29 | Sanitation Standard Operating Procedures (SOPs) | 7 |
| 30 | Inspección ante-mortem y post-mortem | 20 |
| 31 | Inspección post-mortem. | 9 |
| 32 | Transport guide | 1 |
| 33 | Sampling Plan | 8 |
| 34 | Cold chain and storage of meat and meat products. | 2 |
| Total items evaluated | | 202 |

Appendix 5.3. Aspects and number of items evaluated by the *Hygiene and Quality Management*

(GHYCAL) for animal processing plants

| Structure of the GHYCAL Instrument | | | Total Score |
| --- | --- | --- | --- |
| Set | Number | Number of Items |  |
| I | WATER CONTROL PLAN | 11 | 55 |
| II | CLEANING AND DISINFECTION PLAN | 12 | 60 |
| III | MANIPULATOR TRAINING AND CONTROL PLAN | 14 | 70 |
| IV | PREVENTIVE MAINTENANCE PLAN | 10 | 50 |
| V | PEST CONTROL PLAN AND SURVEILLANCE SYSTEMS | 9 | 45 |
| WE | TRACEABILITY CONTROL PLAN | 13 | 65 |
| VII | WASTE MANAGEMENT PLAN | 8 | 40 |
| TOTAL | | 77 | 385 |
